# miRNA-29a as a tumor suppressor mediates PRIMA-1^Met^-induced anti-myeloma activity by targeting c-Myc

**DOI:** 10.18632/oncotarget.6880

**Published:** 2016-01-11

**Authors:** Manujendra N. Saha, Jahangir Abdi, Yijun Yang, Hong Chang

**Affiliations:** ^1^ Division of Molecular and Cellular Biology, Toronto General Research Institute, Toronto, Ontario, Canada; ^2^ Department of Laboratory Medicine & Pathobiology, University of Toronto, Toronto, Ontario, Canada; ^3^ Department of Applied Chemistry, School of Chemical Engineering and Technology, Tianjin University, Tianjin, P. R. China; ^4^ Department of Laboratory Hematology and Medical Oncology, University Health Network, Toronto, Ontario, Canada

**Keywords:** myeloma, miRNA-29a, Myc, apoptosis

## Abstract

The proto-oncogene c-Myc plays substantial role in multiple myeloma (MM) pathogenesis and is considered a potential drug target. Here we provide evidence of a novel mechanism for PRIMA-1^Met^, a small molecule with anti-tumor activity in phase I/II clinical trial, showing that PRIMA-1^Met^ induces apoptosis in MM cells by suppressing c-Myc and upregulating miRNA-29a. Our study further demonstrates that miRNA-29a functions as a tumor suppressor which targets c-Myc. The baseline expression of miR-29a was significantly lower in MM cell lines and MM patient samples compared to normal hematopoietic cells. In addition, ectopic expression of miRNA-29a or exposure to PRIMA-1^Met^ reduced cell proliferation and induced apoptosis in MM cells. On the other hand, overexpression of c-Myc at least partially reverted the inhibitory effects of PRIMA-1^Met^ or miRNA-29a overexpression suggesting the miRNA-29a/c-Myc axis mediates anti-myeloma effects of PRIMA-1^Met^. Importantly, intratumor delivery of miRNA-29a mimics induced regression of tumors in mouse xenograft model of MM and this effect synergized with PRIMA-1^Met^. Our study indicates that miRNA-29a is a tumor suppressor that plays an important role during PRIMA-1^Met^-induced apoptotic signaling by targeting c-Myc and provides the basis for novel therapeutic strategies using miRNA-29a mimics combined with PRIMA-1^Met^ in MM.

## INTRODUCTION

Multiple myeloma (MM) is a plasma cell malignancy characterized by the aberrant expansion of plasma cells within the bone marrow [1]. Despite recent advances in the introduction of the new drugs including proteasome inhibitors (bortezomib), MM is still an incurable disease for its resistance to current therapies [1–3]. Therefore, development of novel treatment options is urgently required for the treatment of MM patients.

PRIMA-1 is a low-molecular weight compound that can restore wild-type conformation and specific DNA binding of mutant p53, consequently triggering apoptosis in tumour cells carrying mutant p53 [4–8]. We have recently demonstrated through *in vitro* and *in vivo* studies that the more effective methylated form, PRIMA-1^Met^, can display a potent anti-myeloma activity without requiring functional activation of p53, which is associated with activation of p63/73 signaling pathway and down-regulation of c-Myc [9]. However; PRIMA-1^Met^ may function through multiple mechanisms, as Tessoulin et al. recently showed that PRIMA-1^Met^ could trigger cell death in MM cells by depleting the glutathione (GSH) content and inducing reactive oxygen species (ROS) [10].

MicroRNAs (miRNAs) are a class of short noncoding and highly conserved RNAs, approximately 22 bp in size [11]. miRNAs regulate gene expression both at transcriptional and translational levels and act in a wide variety of physiological and biological processes, such as cell proliferation, differentiation, and hematopoiesis [12]. Emerging evidence shows that miRNAs play a critical role in tumor pathogenesis by functioning either as oncogenes or tumor-suppressor genes [13]. We and others have shown that certain miRNAs are deregulated in primary MM or established MM cell lines and play key roles in regulatory networks controlling proliferation and/or survival [14, 15]. However, very little is known about miRNAs involvement in response to small molecule anti-tumor agents, particularly PRIMA-1^Met^/APR246, that has been tested in first-in-human clinical trial in refractory hematological malignancies and prostate cancer [16]. Here we present evidence that miRNA-29a mediates PRIMA-1^Met^-induced cell death in MM by targeting c-Myc and that lipid-based delivery of miRNA-29a mimics displays substantial anti-myeloma activity in MM xenograft model, which synergizes with PRIMA-1^Met^.

## RESULTS

### PRIMA-1^Met^ induces differential expression of tumor suppressor miRNAs in MM cells

**Figure 1:**
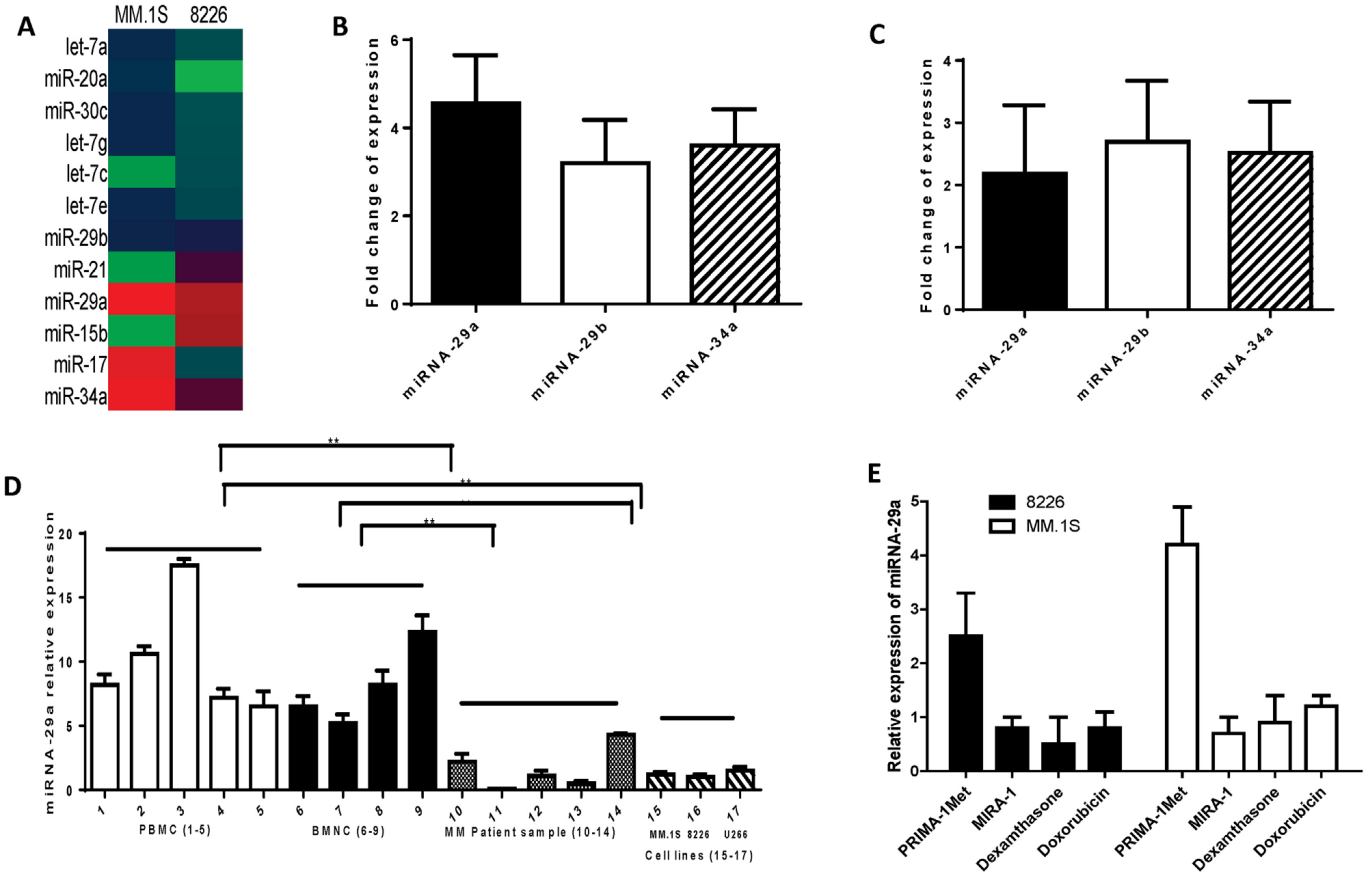
Differential expression of miRNAs between MM cells treated with PRIMA-1^Met^ or DMSO control **A.** MM.S or 8226 cells were treated with PRIMA-1^Met^ (10 or 20 μM, respectively). After 8h cells were harvested to isolate total RNA including miRNA. miRNA was reverse transcribed followed by qPCR array analysis in a 96-well plate targeting the cancer pathway finder (MM.1S) or apoptosis pathway (8226). Data were analysed by the online software (SABiosciences) to see the differential expression of the miRNAs. **B and C.** cDNAs were further used to validate the expression of miRNAs (miRNA-29a, miRNA-29b, and miRNA-34a) in MM.1S (B) and 8226 (C) cells. Fold-changes of the genes are shown after normalizing the data with to a set of housekeeping genes. **D and E.** miRNA-29a expression in MM patient samples and normal hematopoietic cells. (D) miRNA-29a expression is significantly higher in normal peripheral blood mononuclear cells (PBMC) or bone marrow mononuclear cells (BMNC#1) compared to MM patient samples and cell lines. RNAs were isolated from purified patient MM cells, PBMCs, and BMNCs from healthy donors, and the cultured MM cell lines, followed by analysis of basal expression level of miRNA-29a using qRT-PCR. Raw Ct values were normalized to housekeeping SNORD61 and relative expression was calculated using the comparative Ct methods. Results shown are means ±SD. (E) MM.1S and 8226 cells were treated with PRIMA-1^Met^ (10 and 20 μM, respectively), MIRA-1 (10 and 20 μM, respectively), dexamethasone (5 μM), or doxorubicin (5 μM). Control cells were treated with DMSO. Eight hours after treatment, cells were harvested for RNA isolation and expression of miRNA by qPCR as described earlier.

The role of miRNAs in mediating small molecule and drug response is not well described. Therefore, we sought to determine whether PRIMA-1^Met^ might alter the expression of miRNAs that were functionally important. For this purpose, the expression of 84 miRNAs targeting both cancer and apoptosis pathways was assessed in two MM cell lines, 8226 and MM.1S, by using miScript miRNA PCR array (Qiagen). Treatment of 8226 and MM.1S cell lines with PRIMA-1^Met^ (20 and 10 μM, respectively) for 8h modulated the expression of a significant number of miRNAs most of which were found to be up-regulated. miRNA-29a/b and miRNA-34a were among the up-regulated miRNAs in response to PRIMA-1^Met^ treatment (Figure 1A). To further validate the miRNA array data, we examined the expression of these three selected miRNAs in above two cell lines after exposure to PRIMA-1^Met^ using the miScript PCR system with specific miScript primer assays for miRNA-29a/b and miRNA-34a. qPCR re-analysis confirmed PRIMA-1^Met^-induced expression of above miRNAs in MM.1S and 8226 cells (Figure 1B and C).

### Analysis for expression of miRNA-29a in normal hematopoietic cells, MM cell lines and MM patient samples

Next, we examined the basal level expression of miRNA-29a in CD138+ MM patient samples and MM cell lines and compared those with normal hematopoietic cells. qPCR analysis revealed that the expression level of these miRNAs was significantly lower in MM cell lines and patient samples compared with normal hematopoietic cells (Figure 1D). These results suggest that low expression of these miRNAs may play a role in progression of the disease. In addition, to examine whether up-regulation of miRNA-29a is specific to PRIMA-1^Met^, we evaluated the effect of other anti-myeloma agents (MIRA-1, dexamethasone, and doxorubicin) on the expression of these miRNAs. In contrast to PRIMA-1^Met^, none of these agents triggered induction of miRNA-29a (Figure 1E) suggesting that increased expression of the miRNA-29a was specific to PRIMA-1^Met^.

### miRNA-29a regulates cell viability and apoptosis in MM cells

**Figure 2:**
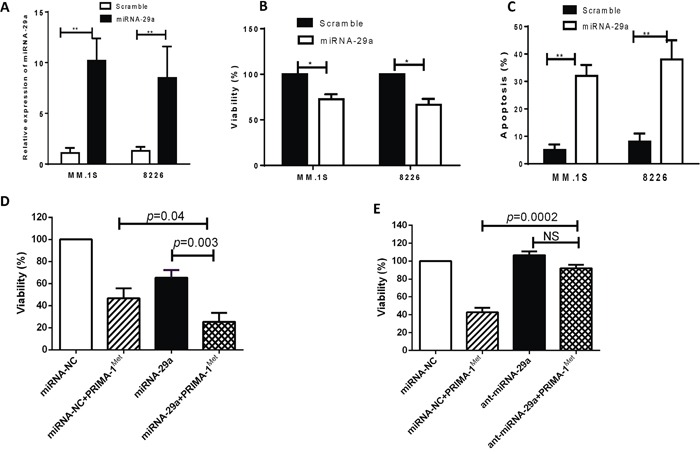
Overexpression of miRNA-29a in MM cells resulted in decrease of cell viability and increase of apoptosis MM.1S or 8226 cells were transiently transfected with either pre-miRNA-29a or scrambled miRNA using the HiPerFect tranfection reagent. **A.** Forty-eight hours after transfection, RNA was isolated from cells transfected with scrambled or miRNA-29a and miRNA expression was analyzed by qPCR. **B and C.** The transfected cells were examined for cell viability and apoptosis by MTT (B) and Annexin V binding assay (C). Results shown are means ±SD (*p<0.05, **p<0.01). **D and E.** Effect of miRNA-29a overexpression or inhibition on PRIMA-1^Met^-induced cytotoxic response in MM cells. MM.1S cells were transiently transfected with either miRNA-29a expression plasmid (D) or synthetic inhibitor of miRNA-29a (E) along with their negative control. Twenty four hours after transfection cells were further treated with PRIMA-1^Met^ and viability of the cells was measured by MTT assay 48h after treatment.

To determine the functional role of miRNA-29a, we overexpressed miRNA-29a in MM cells and investigated its growth inhibiting activity. To this end, we transfected MM.1S and 8226 cells, that constitutively express relatively low level of this miRNA, with pre-miRNA-29a or miRNA-NC. Successful transfection is indicated by significant increase of miRNA-29a expression in miRNA-29a-transfected cells (Figure 2A). Moreover, ectopic expression of miRNA-29a triggered significant inhibition of cell viability (Figure 2B) and increased apoptosis (Figure 2C) in both cells. To investigate the role of miRNA-29a in response to PRIMA-1^Met^, we examined the effect of PRIMA-1^Met^ in MM cells overexpressing miRNA-29a. To this aim, MM.1S cells were transfected with pre-miRNA-29a or miRNA-NC and then treated with PRIMA-1^Met^ (10 μM) or DMSO for 48 h. Viability of the transfected cells was determined as explained above. Treatment of miRNA-29a-transfected MM cells with PRIMA-1^Met^ resulted in significant inhibition of cell viability compared with the cells treated with PRIMA-1^Met^ alone (Figure 2D). Using the Chou-Talalay method as described previously [17], we found the combinatory effect of miRNA-29a and PRIMA-1^Met^ was synergistic (CI= 0.92). To further examine if the miRNA-29a regulate the cytotoxic response of MM cells to PRIMA-1^Met^, we transiently transfected MM.1S cell lines with synthetic inhibitor of AMO-29a. The transfected MM cells were treated with 10 μM PRIMA-1^Met^ for 48h. Viability was measured as described above. Inhibition of miRNA-29a expression reversed the growth inhibitory activity of PRIMA-1^Met^ (Figure 2E) suggesting an important role of miRNA-29a in sensitizing MM cells to PRIMA-1^Met^.

### PRIMA-1^Met^ down-regulates transcriptional and translational expression of c-Myc in MM cells

**Figure 3:**
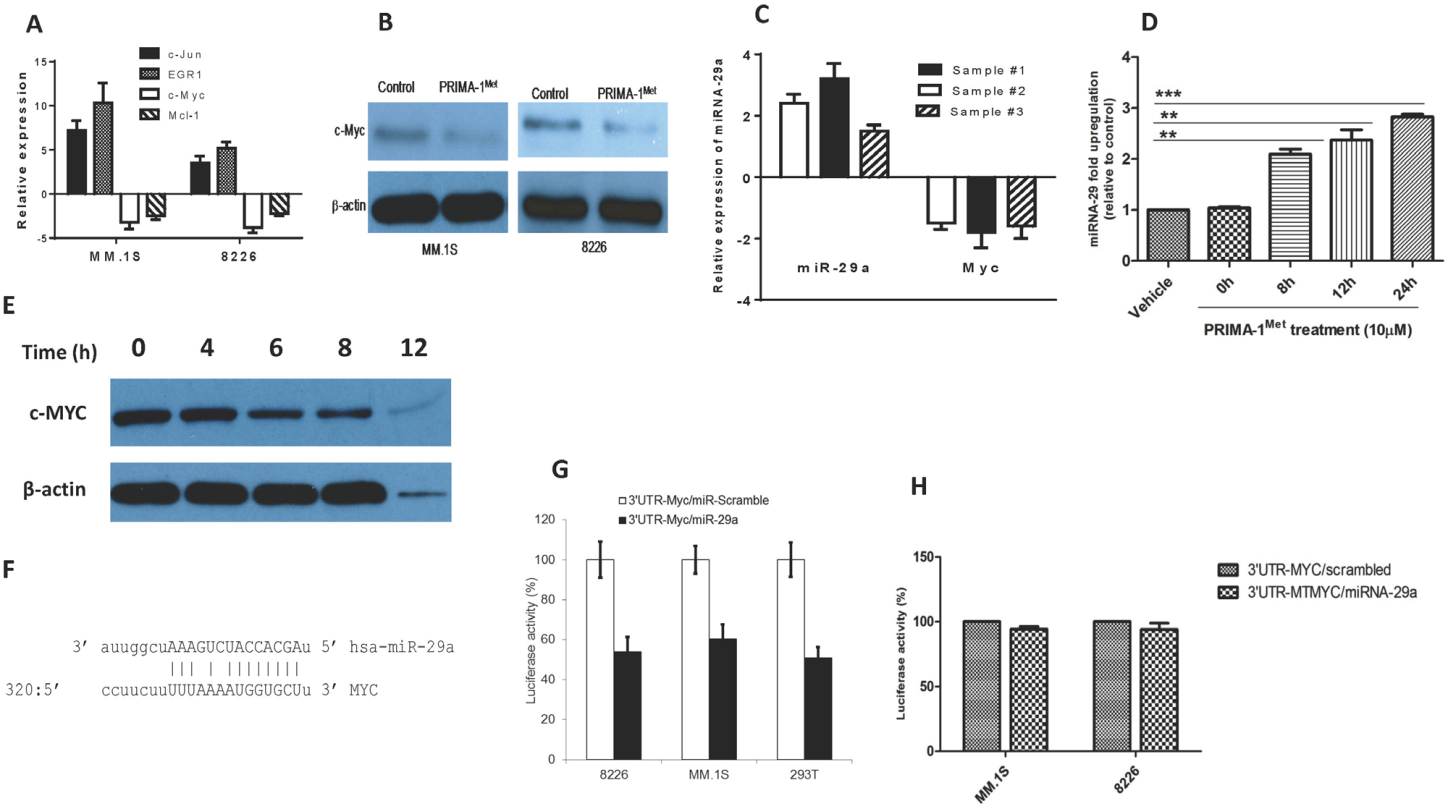
PRIMA-1^Met^ down-regulates the transcriptional and translational expression of c-Myc in MM cells **A.** MM.1S and 8226 cells were treated with 10 or 20 μM PRIMA-1^Met^ for 8h. Cells were harvested for RNA isolation and cDNA synthesis. Resulting cDNA was analysed for the expression of c-Myc and other genes by qPCR. **B.** MM.1S and 8226 cells were treated with PRIMA-1^Met^ as indicated above after which total proteins were isolated for WB analysis. **C.** Primary MM samples obtained from 3 MM patients were treated with 20 μM PRIMA-1^Met^ and expression of miRNA-29a and c-Myc was analysed by qPCR. **D and E.** Time course analysis of miRNA-29a (D) and c-Myc protein (E) after treatment with 10 μM PRIMA-1^Met^ in MM.1S cells. **F-H.** c-Myc as a target for miRNA-29a. **(F)** Sequence alignment of the miRNA-29a seed sequence with c-Myc 3′-UTR. Matched nuclear acid base pairs were linked as “ - ”. **(G, H)** MM.1S and 8226 cells were transiently co-transfected with the reporter plasmids (pEZX-MT-Control, pEZX-3′-UTR/MYC-WT, or pEZX-3′-UTR/MYC-MT) and miRNAs (miRNA-29a or scrambled control miRNA). Cells were harvested and lysed 48h after transfection, followed by measurement of relative fluorescence intensity of firefly and Renilla luciferase according to manufacturer's instruction. Luciferase activities were analyzed as relative activity of firefly to Renilla. Results shown are means ± SD. *p<0.05.

In light of the fact that c-Myc is a critical player in MM oncogenesis [18] and, more importantly, has been shown to suppress miRNA-29 family members through genetic and epigenetic mechanisms [19, 20], we sought to explore whether PRIMA-1^Met^ could also affect c-Myc expression. Both qPCR and Western blot analysis showed that PRIMA-1^Met^ treatment decreased c-Myc expression in MM.1S and 8226 cells (Figure 3A–B). Opposite changes in expression level between miRNA-29a and c-Myc was also observed in 3 different primary MM samples in response to 20 μM PRIMA-1^Met^ (Figure 3C). In addition, time-course experiments were performed for the effect of 10 μM PRIMA-1^Met^ on miRNA-29a and c-Myc protein in MM.1S cell line. miRNA-29a was increased but c-Myc protein decreased in a time-dependent manner. However, non-specific toxic effects of PRIMA-1^Met^ were observed beyond 8h (as indicated by reduced actin band) (Figure 3D, E). These findings imply that PRIMA-1^Met^-induced downregulation of c-Myc and upregulation of miRNA-29a might be inversely related. Additionally, transfection of AMO-29a in MM.1S cells antagonized downregulation of c-Myc at gene and protein levels upon PRIMA-1^Met^ treatment further supporting that PRIMA-1^Met^ down-regulates c-Myc through interaction with miRNA-29a (Supplementary Figure 1).

### c-Myc as a target for miRNA-29a

Having shown that PRIMA-1^Met^ alters c-Myc and miRNA-29a expression in opposite manners, we next tried to understand whether c-Myc could be a target for miRNA-29a. Bioinformatics analysis using MiRanda indicated that the Myc 3′-UTR harbors one target for miRNA-29a (12-14). This site is at least partially complementary to a heptamer motif that is found in the seed region of the miRNA-29a (Figure 3F). To validate c-Myc as a direct target of miRNA-29a, we cloned the 3′ UTR sequence of human c-Myc into the luciferase-expressing vector pEZX-MT01 to the downstream of the luciferase stop codon. miRNA-29a significantly reduced luciferase activity compared with the scrambled control miRNA in MM.1S or 8226 MM cells and 293T cells (Figure 3G) indicating that miRNA-29a binds to the 3′UTR of c-Myc and impairs its mRNA translation. On the other hand, luciferase activity of the mutant 3′UTR clone of c-Myc did not show any change in the presence of miRNA-29a further confirming c-Myc as a direct target of this miRNA (Figure 3H).

### c-Myc plays substantial role in miRNA29a-mediated PRIMA-1^Met^-induced cytoxic response on MM cells

**Figure 4:**
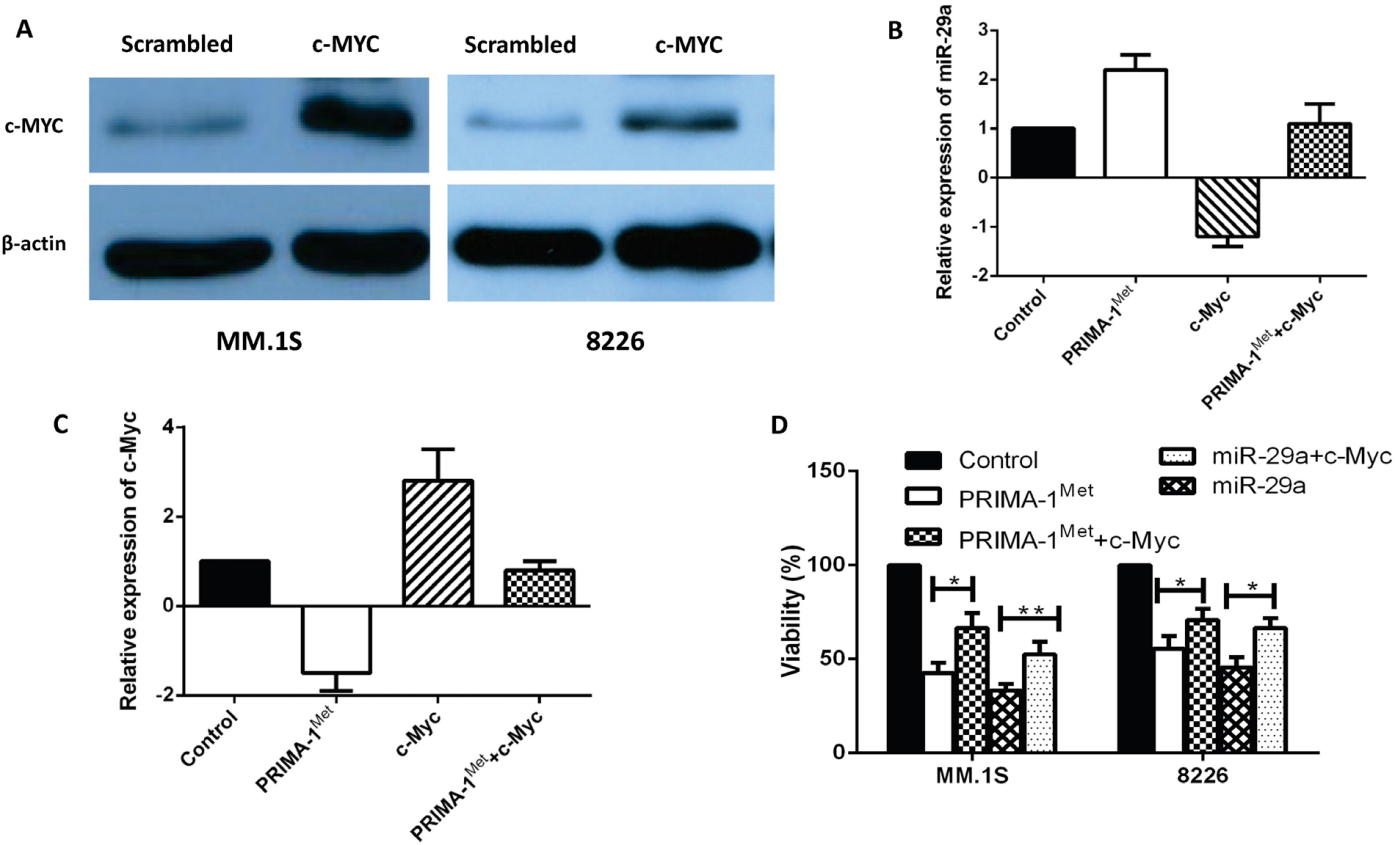
Overexpression of c-Myc reverted apoptosis and cell viability induced by miRNA-29a in MM cells MM.1S and 8226 cells were transiently transfected with expression plasmid of c-Myc. **A.** Overexpression of c-Myc was validated by western blotting in both cell lines. **B, C.** Inverse co-relationship between miRNA-29a and c-Myc was confirmed in MM.1S cell line by overexpression of c-Myc or treatment with PRIMA-1^Met^. **D.** Cells were then analysed for cell viability using the MTT assay. Results shown are representative of means ±SD (*n* = 3). *P<0.05, ** *P*<0.01.

**Figure 5:**
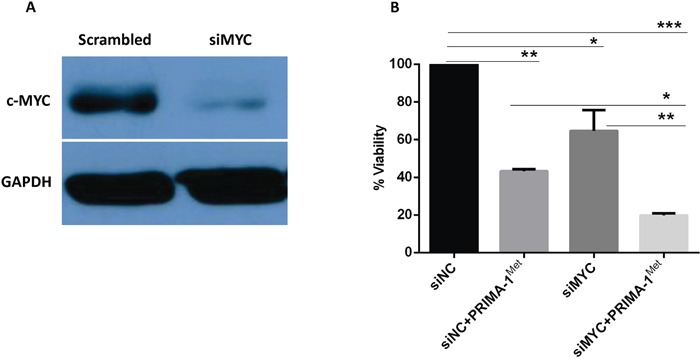
Silencing of MYC gene triggers MM cells death and potentiates PRIMA-1^Met^-induced cytotoxicity MM.1S cells were transfected with 15 pico-mole of MYC siRNA using Lipofectamine RNAiMAX transfection reagent. Twenty four hours after transfection, cells were treated with 10 μM PRIMA-1^Met^ for further 48h and viability was determined with MTT assay. **A.** Validation of c-Myc protein knocking-down in western blotting. **B.** Viability testing using MTT assay. Results shown are representative of means ±SD (*n* = 3). *P<0.05, ** *P*<0.01,*** *P*<0.001

To establish a functional link between c-Myc and miRNA-29a, c-Myc was overexpressed in MM cell lines. MM.1S or 8226 cells were transiently transfected with expression plasmid of c-Myc, or co-transfected with expression plasmid of c-Myc, miRNA-29a or negative control and cells were analyzed for analysis of miRNA-29a, c-Myc and cell viability. Over-expression of c-Myc in MM.1S and 8226 cells was first verified by Western blotting (Figure 4A) and then relative expression of miRNA-29a and c-Myc in MM.1S cells was measured by qPCR assay (Figure 4B, C). Inhibition of viability induced by overexpression of miRNA-29a or PRIMA-1^Met^ treatment was prevented following overexpression of c-Myc in both MM.1S and 8226 cells (Figure 4D). These results indicate that over expression of c-Myc reverts the effect of miRNA-29a in MM cells. To further evaluate the impact of c-Myc in PRIMA-1^Met^-mediated cell death, we silenced c-Myc gene in MM.1S cells followed by measurement of miRNA-29a level and cell viability in the presence or absence of PRIMA-1^Met^. Silencing of c-Myc significantly induced cell death and further enhanced the cytotoxic effects of PRIMA-1^Met^ (Figure 5A, B).

### *In vivo* anti-myeloma activity for the combination of miRNA-29a and PRIMA-1^Met^

**Figure 6:**
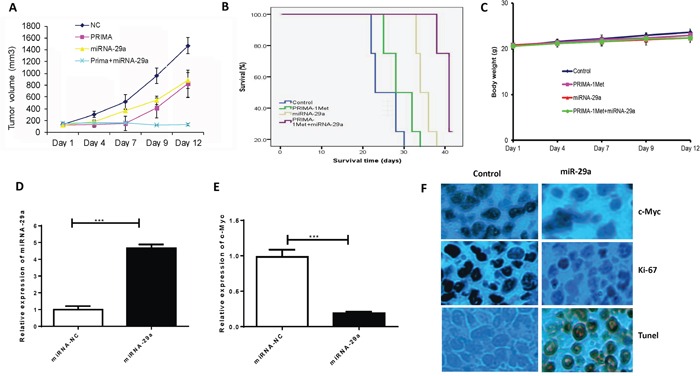
Lipid based delivery of synthetic miRNA-29 mimics retarded tumor growth and prolonged survival in human MM xenograft models 8226 (5 × 10^6^) cells were subcutaneously injected into SCID mice (5mice/group). Administration of miRNA was performed via intratumor injection (MaxSuppressor *in vivo* RNA Lancer II, BIOO Scientific, Austin, TX) of miRNA-29a or scrambled control oligos for a total of 5 injections in 3-day intervals. **A.** miRNA-29a inhibited tumor growth *in vivo*. **B.** Survival was evaluated using Kaplan-Meier curves and log-rank analysis from the first day of tumor cells injection until death or occurrence of an event. **C.** Body weight was measured every three-day till day 39 and data were presented as means ±SD. **D and E.** Mice tumors from the experiment described in this panel were analyzed by qPCR for miRNA-29a (D) and c-Myc (E) expression. **F.** Immunohistochemical analysis of tumor sections showed that treatment of mice bearing tumors of 8226 with miRNA-29a overexpression resulted in decrease in the proliferation index (Ki-67), c-Myc and increase in the apoptotic index (TUNEL), further verifying the targeting of c-Myc by miRNA-29a to induce apoptosis in MM cells *in vivo*.

To further study the potential therapeutic applicability of our findings as a novel anti-MM treatment, we examined the effect of miRNA-29a in combination with PRIMA-1^Met^ in MM xenograft model. When tumors became measurable, mice were assigned into 4 groups (5 mice/group). One group of mice was injected with PRIMA-1^Met^ at 50 mg/kg given intraperitoneally (i.p.). miRNA was delivered at a lower concentration (10μg) by lipid-based methods as explained above. Another group was treated with combination of PRIMA-1^Met^ and miRNA-29a mimics. Control group was treated with non-targeting miRNA and PBS. Treatments were performed once daily for 14 days. Tumor growth, body weight, and survival of the mice were monitored. Co-treatment of PRIMA-1^Met^ and miRNA-29a mimics resulted in significant inhibition of tumor growth (Figure 6A) and prolongation of survival (Figure 6B) in mice compared to the mice treated with either PRIMA-1^Met^ or miRNA-29a mimics or vehicle. No significant changes in the body weight of the mice in these groups was observed indicating that doses of the miRNA-29a mimics or PRIMA-1^Met^ were tolerable (Figure 6C). The high expression of miRNA-29a and low expression of c-Myc observed by qPCR in tumor samples of the miRNA-29a-treated mice (Figure 6D–E) validate successful delivery of miRNA-29a mimics. The expression of Ki-67 and c-Myc decreased and that of TUNEL increased in tumor samples from miRNA-29a-mice compared to samples obtained from the vehicle-treated mice (Figure 6F).

## DISCUSSION

The small molecule PRIMA-1^Met^ has proved to be pre-clinically and clinically effective in MM and other tumors [4–8]. In MM, some reports including our previous work have disclosed to some level the mechanisms underlying PRIMA-1^Met^-induced cell death [9]. However, regarding the potential roles oncomiRNAs and tumor suppressor miRNAs play in MM pathogenesis [21], it's not known whether PRIMA-1^Met^ imposes its anti-myeloma activities through miRNAs. Our study revealed significant lower expression of miRNA-29a in MM cell lines and patient primary cells compared to normal hematopoietic cells, enforced expression of miRNA-29a inhibited viability and triggered apoptosis in MM cells *in vitro*. miRNA-29a was upregulated in MM cells following PRIMA-1^Met^ treatment leading us to further explore the role of miRNA-29a as a possible tumor suppressor in PRIMA-1^Met^-induced effects.

It is well established that miRNA-oncogene/tumor suppressor axis plays substantial role in drug response, growth and proliferation of cancer cells [22], however; whether such an axis operates to mediate the PRIMA-1^Met^-induced effects is unknown. Interestingly, our study revealed that miRNA-29a mediated PRIMA-1^Met^-induced cell death in MM cells by targeting c-Myc, a mechanistic concept that has not been explored for PRIMA-1^Met^ in MM before. We showed that PRIMA-1^Met^ treatment of MM cell lines and MM primary cells upregulated miRNA-29a and downregulated c-Myc. PRIMA-1^Met^ increased miRNA-29a-induced cytotoxicity in MM cells, whereas miRNA-29a inhibition by its synthetic inhibitor attenuated PRIMA-1^Met^-induced inhibition of viability suggesting that miRNA-29a mediates anti-myeloma activity of PRIMA-1^Met^. Further, inhibition of cell proliferation and induction of apoptosis by PRIMA-1^Met^ at concentrations tolerable in MM [9, 10, 16], were associated with down-regulation of c-Myc and up-regulation of miRNA-29a. Our *in vitro* observations yield evidence that miRNA-29a-Myc axis could function, at least partly, as a novel mechanism to regulate anti-myeloma activity of PRIMA-1^Met^. In line with our findings, a recent report indicates that some miRNAs may be involved in MM cells’ response to bortezomib, however; potential oncogenes or tumor suppressors linked to these miRNAs requires continuous exploration [23].

**Figure 7:**
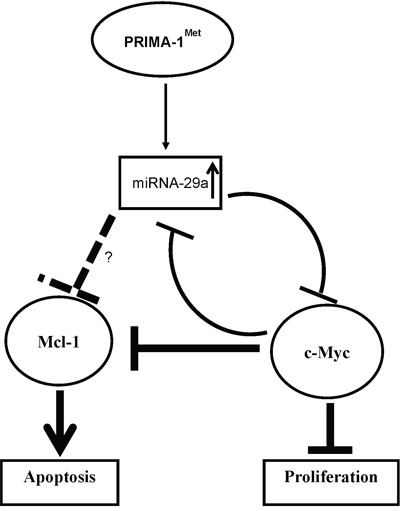
Scheme of proposed mechanism for regulation of c-Myc by miRNA on PRIMA-1^Met^ treatment miRNA-29a-mediated targeting of c-Myc by PRIMA-1^Met^ may occur as a direct inhibitory effect of the miRNAs. Alternatively, such inhibition can be seen as an indirect effect of the miRNAs which may be mediated by other targets including Mcl-1 or Bcl-2.

In our study, c-Myc was downregulated by miRNA-29a enforced expression or PRIMA-1^Met^ treatment. On the contrary, in the presence of miRNA-29a inhibitor c-Myc transcript or protein did not change and PRIMA-1^Met^-induced suppression of c-Myc was at least partially reversed. Additionally, overexpression of c-Myc reduced miRNA-29a level and antagonized PRIMA-1^Met^–induced MM cell death. This is consistent with the report that c-Myc is part of a multi-component regulatory complex which trans-repress several miRNAs including miRNA-29a [24]. Our findings suggest that miRNA-29a-Myc axis mediates the effects of PRIMA-1^Met^ in MM cells through a feedback loop mechanism (Figure 7). However; we did not observe any significant change at miRNA-29a level following c-Myc silencing (data not shown). It is possible that other factors in the repressor complex might have compensated, or knockdown of other components in addition to c-Myc in the regulatory complex might be needed, more extensive experiments are required to address this issue. Since c-Myc plays a critical role in MM pathogenesis as a strong promoter of MM cell growth and survival [25], we found that knocking down of c-Myc reduced MM cell viability and sensitized them to PRIMA-1^Met^–induced toxicity.

Several studies demonstrate a mutual functional link between c-Myc and some Bcl-2 family-related proteins including Mcl-1 [26–28]. For instance, GP Gregory et al. showed that dinaciclib (CDK9 inhibitor) suppressed both c-Myc and Mcl-1 and inhibited progression of c-Myc-driven lymphoma in mice xenograft models [28]. We previously showed that PRIMA-1^Met^ could induce cell death in MM cells by suppressing Mcl-1 but the mediator (s) of this effect was not defined [9]. In the present study, PRIMA-1^Met^ downregulated Mcl-1 transcript and protein (Figure 4A, supplementary figure 1), and interestingly this effect was partially reversed by AMO-29a in MM.1S cells suggesting that PRIMA-1^Met^ may also trigger MM cell death through miRNA-29a targeting Mcl-1. Indeed miRNA-29a/b have previously been shown to be associated with targets involved in regulation of apoptosis such as Bcl-2 and Mcl-1 that are often deregulated in malignant cells [29–32]. Of note, apoptosis-inducing effects of miRNA-29b have been demonstrated in MM cells by several studies indicating the potential of miRNA-29 family members mostly as tumor suppressors in MM [30, 33]. Furthermore, since PRIMA-1^Met^ suppressed both Mcl-1 and c-Myc leading to apoptosis in MM cells in our system (Figure 7), it is speculated that the two proteins could also interact in response to PRIMA-1^Met^ building a network with miRNA-29a to control PRIMA-1^Met^-induced cell death in MM. It is conceivable that suppression of c-Myc (following enforced expression of miRNA-29a or PRIMA-1^Met^ treatment) may induce apoptosis of MM cells directly or through inhibition of Mcl-1.

Finally, our study shows that injection of miRNA-29a synthetic mimics inhibits tumor growth and prolongs survival in subcutaneous mouse xenograft model and this effect is enhanced in combination with PRIMA-1^Met^ indicating that our *in vitro* observations can be successfully translated *in vivo*. Our data are in agreement with reports by Trang and colleagues [34] and Di Martino et al. [35] on the safe use of formulated NLE-miRNAs in experimental animals, and strongly support clinical development of miRNA-29a-based therapeutic strategies in MM patients. Notably, formulated miRNA mimics are distinct from molecularly targeting drugs whose antitumor activity relies on the modulation of a wide range of genes rather than inhibition of individual gene products [34–36].

## MATERIALS AND METHODS

### Cell culture and drug treatment

The MM cell lines, MM.1S, RPMI-8226 (8226), NCI-H929, U266, and LP1 were cultured in complete RPMI-1640 medium supplemented with 10% FBS, 100 units/mL penicillin, 100 μg/mL streptomycin, and 2mM L-glutamine. NCI-H929, U266, and MM.1S cell lines were obtained from American Type Culture Collection (ATCC). CD138+ cells were freshly isolated and purified from MM patients as described previously [9]. PBMCs and bone marrow mononuclear cells (BMNCs) were collected from healthy donors and maintained in the culture medium as described previously [9]. This study was approved by the research ethics committee of University Health Network, Toronto, Canada, in accordance with declaration of Helsinki.

### RNA extraction, miRNA array, and real-time PCR

Total RNA or miRNA was extracted using RNeasy kit (Qiagen) or miRNA Easy kit (Qiagen). RNA quantity was determined with a spectrophotometer (ND-1000; Nano-Drop Technologies). cDNAs were synthesized using miScript II RT kit (QIAGEN) and applied to Cancer Pathway Finder or Apoptosis miRNA array plates (SABiosciences) for miRNA measurement using miScript SYBR Green PCR kit (QIAGEN) in an ABI-7900HT machine following manufacturer's instructions. The expression of mature miRNAs was calculated relative to SNORD72. The data were analyzed by RT^2^ Profiler PCR Array Data Analysis v3.5, and fold-changes in miRNA treatments relative to scrambled treatments were calculated by using the 2^−ΔΔCt^ algorithm.

### Overexpression of miRNA-29a, c-Myc and inhibition of miRNA-29a in MM cells

MM.1S or 8226 cells were transiently transfected with either pre-miRNA-29a, c-Myc expression plasmid or scrambled miRNA (Gene Copoeia) using the HiPerFect transfection reagent (Qiagen). The transfected cells were then treated with PRIMA-1^Met^ or DMSO and examined for cell viability using the 3-(4,5-dimethylthiozol-2-yl)-2,5-diphenyl tetrazolium bromide (MTT; Biobasic) or apoptosis assays 48h after transfection. In other experiments, the above cell lines were transfected with synthetic miRNA-29 inhibitor (AMO-29a) using HiPerFect reagent and treated with PRIMA-1^Met^.

### Apoptosis assays

An Annexin V–FITC/propidium iodide apoptosis detection kit (BD Biosciences) was used to quantify apoptosis in a FACS Calibur flow cytometer (BD Biosciences) and data were analyzed using CellQuest software (BD Biosciences).

### c-Myc silencing and its effects on PRIMA-1^Met^-induced cytotoxicity

MM.1S cell line was transfected with 15 pico-mole of c-Myc siRNA (Ambion Silencer Select, Life Technologies) using Lipofecatmine RNAiMAX. Knocking-down of the protein was confirmed with Western blotting. Additionally, 24h after transfection cells were treated with 10μM PRIMA-1^Met^ and incubated for additional 24 h. Viability of cells was determined using MTT assay as described above.

### Protein extraction and western blotting analysis

Cells were lysed in cold cell lysis buffer (50mM Tris-HCl, pH 7.4, 150mM NaCl, 1% NP-40, 0.5% sodium deoxycholate, and 0.1% SDS) supplemented with protease inhibitor cocktail (Complete mini, Roche). Equal amounts of proteins were resolved by 12% SDS-PAGE and transferred onto PVDF membranes. Membranes were blocked by incubation in 5% nonfat dry milk in PBST (0.05% Tween-20 in PBS) and probed with anti-Myc, anti-Mcl-1 and anti-GAPDH (Signalway Antibody, Baltimore, MD, USA). Blots were then developed by enhanced chemiluminescence kit (Millipore).

### Reporter assay

pEZX-reporter plasmids for the miRNA-29a putative target c-Myc were constructed. 293T cells, MM.1S or 8226 cells were transiently cotransfected with reporter plasmids (pEZX-MT-Control, or pEZX-3′-UTR/c-Myc), miRNA-29a or control miRNA using HiPerFect transfection reagent. Cells were harvested 48 h after transfection and lysed, then firefly and Renilla luciferase activities were measured using the Dual-Luciferase Reporter Assay System following manufacture's instruction. Luciferase activities were analyzed as the activity of firefly relative to Renilla.

### *In vivo* models of human MM

Severe combined immuno-deficient (SCID) mice (6 to 8 weeks old; OCI) were housed and monitored in our Animal Research Facility. All experimental procedures and protocols had been approved by the Institutional Ethical Committee (University Health Network, Toronto). To establish subcutaneous human MM xenograft models, 5 × 10^6^/100μl PBS of 8226 cells were mixed in 100μl Matrigel matrix (BD Biosciences) and injected subcutaneously into the right flank of SCID mice (*n* = 5, 5 mice/group). The mice started developing subcutaneous tumors approximately 25 days after injection. Tumor size was monitored and measured every 3 days in 2 dimensions using calipers, and tumor volume was calculated using the following formula: V = 0.5a×b^2^, where “a” and “b” are the long and short diameters of the tumor, respectively. Survival was evaluated from the first day of tumor injection until death. In accordance with institutional guidelines, mice were sacrificed when their tumors reached 1.5 cm in diameter or in the event of paralysis or major compromise in their quality of life, to prevent unexpected suffering.

### Lipid-based delivery of synthetic miRNA

Mice were randomized into 4 groups and treated with synthetic miRNA-29a mimics, miRNA-NC, PRIMA-1^Met^ (50 mg/kg) or the combination of miRNA-29a mimics and PRIMA-1^Met^. Each dose of miRNA contained 20 μg synthetic oligo which equals 1mg/kg per mouse with an average weight of 20g. Administration of miRNA mimics was performed using the novel formulation of neutral lipid emulsion (NLE) (MaxSuppressor *in vivo* RNA Lancer II, BIOO Scientific, Austin, TX) according to the manufacturer's instructions. Treatments were performed intratumorally by using the formulation and dosage as described above. Tumors were then collected and placed in 10% formalin for histologic assessments.

### Statistical analysis

Each experiment was performed at least 3 times and all values are reported as means ±SD. Comparisons between groups were made with student's *t*-test, while statistical significance of differences among multiple groups was determined by GraphPad Prism software. *p* value of less than 0.05 was accepted as statistically significant.

## CONCLUSION/CLINICAL SIGNIFICANCE OF THE STUDY

Our investigation provides evidence on miRNA-29a involvement in PRIMA-1^Met^-induced inhibition of cell proliferation and induction of apoptosis in MM cells, and supports the development of miRNA-29a mimics as potential pharmacological intervention strategy in MM. The important interaction with c-Myc opens new opportunities for combinatory therapeutical approaches which may result in a selective and highly efficient targeting of pathways crucially involved in the control of MM cell growth and survival.

## SUPPLEMENTARY FIGURE


