# Supplementary material for: miRNA-29a as a tumor suppressor mediates PRIMA-1Met-induced anti-myeloma activity by targeting c-Myc
Source: Oncotarget. 2016 Jan 11;7(6):7149–60. doi: 10.18632/oncotarget.6880 (PMC4872775; doi:10.18632/oncotarget.6880)
Supplement: Supplementary file 1 [file oncotarget-07-7149-s001.pdf]

SUPPLEMENTARY FIGURE

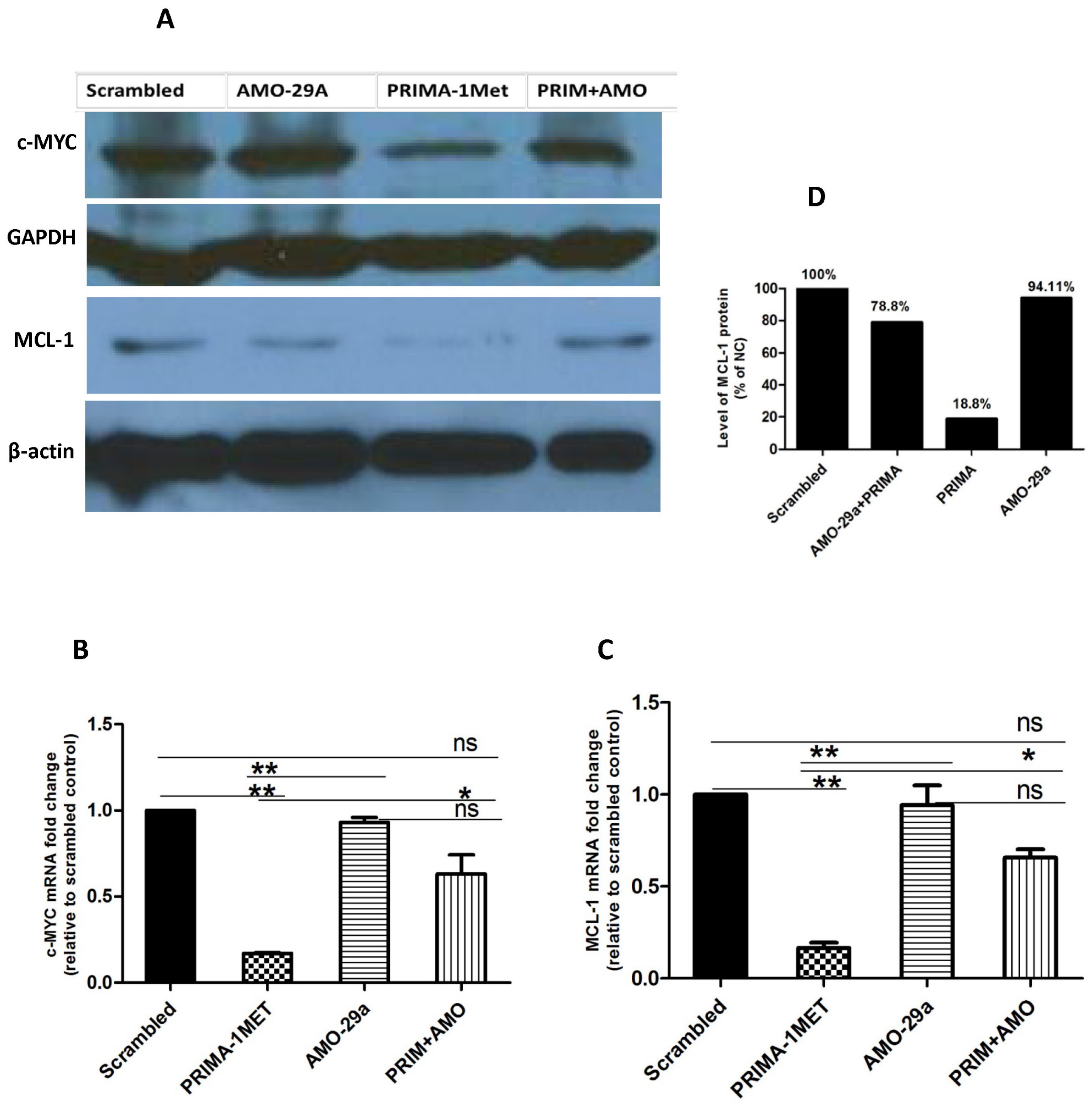

Supplementary Figure S1: Inhibition of miRNA-29a (AMO-29a) reverses PRIMA-1^{Met}-induced c-Myc and Mcl-1 downregulation at protein (**A**) and transcript (**B**) levels. MM.1S cell line was transfected with synthetic AMO-29a using HiPerFect transfection reagent. After 24 h transfected cells were treated with 10µM PRIMA-1Met for further 24 h (protein assessment) or 8 h (qPCR analysis). The data represent mean ± SD from two separate experiments. *p<05, **p<0l. (**D**)-Quantification of MCL-1 bands. Band densities were quantified using imageJ software, ratios of each MCL-1 band to its relevant beta-actin was determined and results were normalized to scrambled control (% of negative control).
